# Dr. E. G. Loring

**Published:** 1888-05

**Authors:** 


THE DEATH OF DR. E. G. LORING.—In the death of Dr. E. G. Loring New York has sustained the loss of another of her most prominent ophthalmologists. He was at one time a partner of Professor C. R. Agnew, and died suddenly of heart disease, just one week after the death of Professor Agnew.

